# An Accurate Model for Biomolecular Helices and Its Application to Helix Visualization

**DOI:** 10.1371/journal.pone.0129653

**Published:** 2015-06-30

**Authors:** Lincong Wang, Hui Qiao, Chen Cao, Shutan Xu, Shuxue Zou

**Affiliations:** The College of Computer Science and Technology, Jilin University, Changchun, Jilin, China; Wake Forest University, UNITED STATES

## Abstract

Helices are the most abundant secondary structural elements in proteins and the structural forms assumed by double stranded DNAs (dsDNA). Though the mathematical expression for a helical curve is simple, none of the previous models for the biomolecular helices in either proteins or DNAs use a genuine helical curve, likely because of the complexity of fitting backbone atoms to helical curves. In this paper we model a helix as a series of different but all bona fide helical curves; each one best fits the coordinates of four consecutive backbone C_α_ atoms for a protein or P atoms for a DNA molecule. An implementation of the model demonstrates that it is more accurate than the previous ones for the description of the deviation of a helix from a standard helical curve. Furthermore, the accuracy of the model makes it possible to correlate deviations with structural and functional significance. When applied to helix visualization, the ribbon diagrams generated by the model are less choppy or have smaller side chain detachment than those by the previous visualization programs that typically model a helix as a series of low-degree splines.

## 1 Introduction

Historically helices were proposed as the main secondary structural elements for proteins in 1951 [1] and as the only structural forms for double stranded DNAs (dsDNA) [2] in 1953 through model building using low-resolution X-ray diffraction data well before atomic coordinates could be determined from high-resolution data [3, 4]. However even with the ready availability of many high-resolution structures at present, a biomolecular helix in either a protein or a DNA molecule has rarely been modeled as a series of genuine helical curves likely because of the difficulty to accurately fit the backbone atoms to helical curves though methods [5, 6] have been proposed in the past to compute helical parameters using backbone atoms: N, C_*α*_, CO atoms in a protein or P atoms in a DNA. For example, the p-curve [6] program computes helical parameters from a series of base planes in a DNA or peptide planes in a protein. Similarly none of the previous programs for helix visualization (typically as a ribbon diagram [7]) model a biomolecular helix as a series of truly helical curves. Instead a biomolecular helix is usually approximated with a series of low-degree splines such as Hermite polynomials that pass through backbone C_*α*_ or P atoms [8–20]. Using a series of low-degree splines as a model for a biomolecular helix has several disadvantages such as (1) the model could deviate largely from a genuine helical curve at both the local and global levels, (2) both the true difference between a helical curve and the biomolecular helix and the errors in the model itself contribute to the deviation. Consequently the deviation could not be quantified and further correlated with structural and functional significance, and (3) when applied to molecular visualization the generated helix ribbon diagrams are either choppy (wavy) or the side chains become detached from the diagrams [16].

In this paper, we describe a model that represents a biomolecular helix by a series of different but all bona fide helical curves each one being computed using a newly-developed curve fitting algorithm that searches for a helical curve that best fits the coordinates of four backbone atoms. A helix model composed of a series of helical curves has been previously called a *polyhelix*[21]. A key difference between our model and a *polyhelix* is that the curves in the latter may not fit well to backbone atoms. The representation of a whole helix as a series of helical curves rather than a single one makes it possible to describe accurately the local deviations of the helix from a genuine helical curve. In particular for a protein helix we have defined a new score to quantify its deviations from the standard protein helix(See section 2.2 for a precise definition of the term *the standard protein helix*.) and to further link the deviations with their locations in proteins. When applied to the visualization of a helix as a ribbon diagram, the model’s closeness to a genuine helical curve makes it possible to eliminate choppiness in protein diagrams and to greatly reduce it in DNA diagrams while the minimization of the distance between a backbone atom and its closest point on the diagram achieved by the curve fitting algorithm greatly alleviates the side chain detachment problem. Either choppiness or detachment appears frequently on the helix diagrams drawn by the previous molecular visualization programs [12, 14–20]. In addition both the deviation and correlation could be easily visualized by the helix diagrams generated by our model.

## 2 The Helical Curve Fitting Algorithm and the Helix Model

Our helix model is composed of a series of genuine helical curves. In this section we first outline the curve fitting algorithm that searches for a helical curve that best fits the coordinates of four backbone atoms. We then define a helix score for residue *i* in a protein helix that quantifies the local deviation from the standard protein helix of the helical curve that best fits a quadruple of residues *i*, *i* + 1, *i* + 2 and *i* + 3. Finally we present the model itself. For ease of exposition, we use four successive C_*α*_ atoms as input.

### 2.1 The helical curve fitting algorithm

A general helical curve in three dimensional (3D) space could be represented as:

$$\left[\begin{array}{c} x\\ y\\ z \end{array}]=[\begin{array}{c} x_{0}\\ y_{0}\\ z_{0} \end{array}]+R\;[\begin{array}{c} r\sin t\\ r\cos t\\ pt \end{array}\right]$$ (1)

where **r** = {*x*, *y*, *z*} (In this paper, bold lower-case letters denote 3D vectors and bold capital letters denote either 3D rotation matrices or 3D curves.) is a point on the curve, **r_0_** = {*x*_0_, *y*_0_, *z*_0_} its origin, and **R** the rotation matrix that specifies its helical axis **n** with respect to a coordinate system. The first three helical parameters, radius (*r*), pitch (*p*) and turn angle (*t*), define a standard helical curve, *x* = *r*sin*t*, *y* = *r*cos*t*, *z* = *pt* with its origin at {1.0,0.0,0.0}, its center at {0.0,0.0,0.0} and its helical axis along the + *Z* axis. Together with **n** and **r**_0_ these five parameters completely define a general helical curve. Though *r*, *p*, *t* could be computed directly from the virtual bond length, bond angle and dihedral angle of a quadruple of C_*α*_s [5], no simple analytic expression has been derived for the computation of a helical curve that best fits the coordinates of a quadruple of C_*α*_s, that is, a helical curve that has the minimum RMSD (Δ_*i*_) between the four C_*α*_s and their closest points on the curve. In fact, this minimization (or curve fitting) problem is equivalent to finding the solutions to a high-degree monomial. The complexity of searching for a series of different helical curves that best fit a series of segments of backbone atoms is likely to be the reason why no genuine helical curves have been used to model a biomolecular helix. In the following we describe briefly an efficient algorithmic solution to this minimization problem.

We begin with the computations of *r*, *p* and *t* using previously-derived analytic expressions [5], and denote their values as *r*_*m*_, *p*_*m*_ and *t*_*m*_. Then we proceed as follows to search discretely and exhaustively over two intervals, [*r*_*m*_ − *δ*_*r*_, *r*_*m*_ + *δ*_*r*_] and [*p*_*m*_ − *δ*_*p*_, *p*_*m*_ + *δ*_*p*_], for the *r* and *p* values of a helical curve that best fits the coordinates of a quadruple of C_*α*_s of residues *i*, *i* + 1, *i* + 2 and *i* + 3. Both *δ*_*r*_ and *δ*_*p*_ are user-specified constants.

1. Δ_*i*_ = ∞ *{the initial RMSD}*
2. For each *r* in [*r*_*m*_ − *δ*_*e*_, *r*_*m*_ + *δ*_*r*_]
   For each *p* in [*p*_*m*_ − *δ*_*p*_, *p*_*m*_ + *δ*_*p*_]
    Compute *t* *{the turn angle}*
    Generate a helical curve *{by* Eq 1}
    Best-fit the curve to the four C_*α*_s using singular-value decomposition(SVD) to compute **R**
    If *d*_*q*_ < Δ_*i*_
     Δ_*i*_ = *d*_*q*_
     
*r*_*i*_ = *r*, *p*_*i*_ = *p*, *t*_*i*_ = *t*, **R**_*i*_ = **R**

where *d*_*q*_ is the RMSD between the quadruple of C_*α*_s and their closest points on the helical curve; *r*_*i*_, *p*_*i*_, *t*_*i*_ and **R**_*i*_ are, respectively, the helical parameters and rotation matrix for the helical curve that best-fits the quadruple. Given both *r* and *p* and the distance *d*_*i*, *i* + 1_ between two consecutive C_*α*_s, *t* could be computed as follows: $t=2\text{arcsin}(0.5\sqrt{(d_{i,i+1}^{2}-p^{2})/r})$. Singular-value decomposition (SVD) is applied to compute Δ_*i*_ and **R**_*i*_; and from **R**_*i*_, both **n**_*i*_ and helix center **c**_0_ for this quadruple of C_*α*_s could be calculated. In fact, the SVD step guarantees that the computed helical curve best fits the coordinates of the quadruple of C_*α*_s. The set of five helical parameters and the centers for all the quadruples of consecutive C_*α*_s in a protein chain or Ps in a DNA strand are computed by sliding over its sequence a window of four C_*α*_ or P atoms.

### 2.2 The helix score for a protein helix

Except for the last three residues at the C-terminus of a protein chain, to each residue *i* of a protein sequence is assigned a helix score *h*_*i*_.

$$h_{i}=\frac{{\left(r_{i}-\mu_{r}\right)}^{2}}{2\sigma_{r}^{2}}\times \frac{{\left(p_{i}-\mu_{p}\right)}^{2}}{2\sigma_{p}^{2}}\times \frac{{\left(t_{i}-\mu_{t}\right)}^{2}}{2\sigma_{t}^{2}}\times \frac{\Delta_{i}^{2}}{2\sigma_{\Delta }^{2}}$$ (2)

where *r*_*i*_, *p*_*i*_, *t*_*i*_,Δ_*i*_ are computed as above using a quadruple of C_*α*_s of residues *i*, *i* + 1, *i* + 2 and *i* + 3. The constants *μ*_*r*_, *σ*_*r*_; *μ*_*p*_, *σ*_*p*_; *μ*_*t*_, *σ*_*t*_ and *σ*_Δ_ are respectively the normal distribution parameters for *r*, *p*, *t*,Δ that are determined as follows using the respective data sets for *r*, *p*, *t*,Δ computed over a non-abundant set 𝕊 of 3,287 X-ray structures in the PDB with each of them has a resolution ≤ 2.0Å, a R-factor ≤ 25.0% and at least three helices. We have applied the program dssp [22] to assign a total of 44,456 helices for the protein structures in 𝕊. The three parameters *μ*_*r*_, *μ*_*p*_ and *μ*_*t*_ define a standard helical curve that represents an average over all the protein helices in 𝕊. For ease of reference, we call it *the standard protein helix*. The term $\frac{\Delta^{2_{i}}}{2\sigma^{2_{\Delta }}}$ quantifies the spatial difference between a C_*α*_ atom and its closest point on the model while the helix score itself determines the deviation of the model from the standard protein helix: the higher the score the larger deviation from the standard protein helix.

### 2.3 The helix model

**Fig 1:**
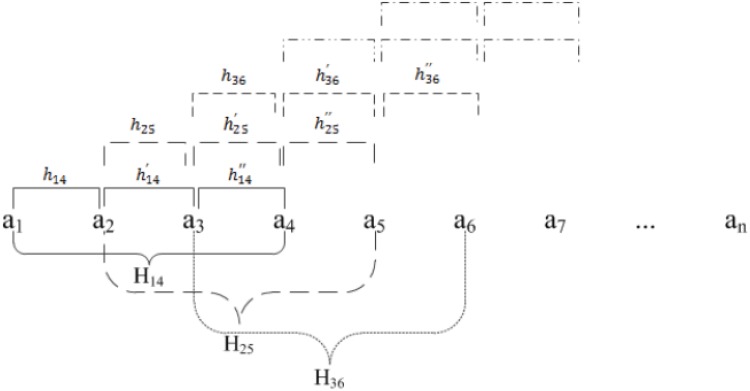
The averaging process for helix model computation. In this example the first helical curve **H**_14_ is computed using the first quadruple of backbone atoms {**a**_1_,**a**_2_,**a**_3_,**a**_4_}, the second curve **H**_25_ the next quadruple of atoms {**a**_2_,**a**_3_,**a**_4_,**a**_5_} and so on. For a pair of two consecutive interior atoms up to three slightly different curves could be computed. The final model curve for the segment between a pair of consecutive atoms is their average (Eq 3).

The model is computed through an averaging process that merges into a single curve all the helical curves for the quadruples of backbone atoms obtained by sliding over a protein helix or a DNA strand. Starting with either the N-terminus of a protein helix or 5’-terminus of a DNA strand, the model curve **c**_*a*_*i*_*a*_*i* + 1__ between two consecutive atoms, **a**_*i*_ and **a**_*i* + 1_, is computed as follows.

$$\left[\begin{array}{c} c_{a_{1}a_{2}}\\ c_{a_{2}a_{3}}\\ c_{a_{3}a_{4}}\\ c_{a_{4}a_{5}} \end{array}]=[\begin{array}{c} h_{14}\\ (h_{14}^{\prime }+h_{25})/2\\ (h_{14}^{\prime \prime }+h_{25}^{\prime }+h_{36})/3\\ (h_{25}^{\prime \prime }+h_{36}^{\prime }+h_{47})/3 \end{array}\right]$$ (3)

where {**a**_1_,**a**_2_,**a**_3_,**a**_4_,**a**_5_,…,**a**_*n*_} denotes *n* consecutive backbone atoms along a protein helix or a dsDNA strand, and **h**_*i*, *i* + 1_ is the segment between two consecutive atoms, **a**_*i*_ and **a**_*i* + 1_, of the helical curve **H**_14_ that is computed using a quadruple of atoms {**a**_1_,**a**_2_,**a**_3_,**a**_4_}, $h_{i,i+1}^{\prime }$ the segment of **H**_14_ between **a**_*i* + 1_ and **a**_*i* + 2_, and $h_{i,i+1}^{\prime \prime }$ the segment of **H**_14_ between **a**_*i*+2_ and **a**_*i*+3_. Please see Fig 1 for an illustration.

## 3 Results and Discussion

We have applied the model to both the protein and DNA molecules to assess its accuracy for the representation of biomolecular helices. In this section in addition to presenting the computational results we also compare our model with and discuss its advantages over the previous helix models for (1) the quantification of the deviation from the standard protein helix of a helix in a protein, (2) the correlation between the deviations and their locations in proteins, and (3) the visualization of helices as ribbon diagrams.

### 3.1 The accuracy of the helical model

**Fig 2:**
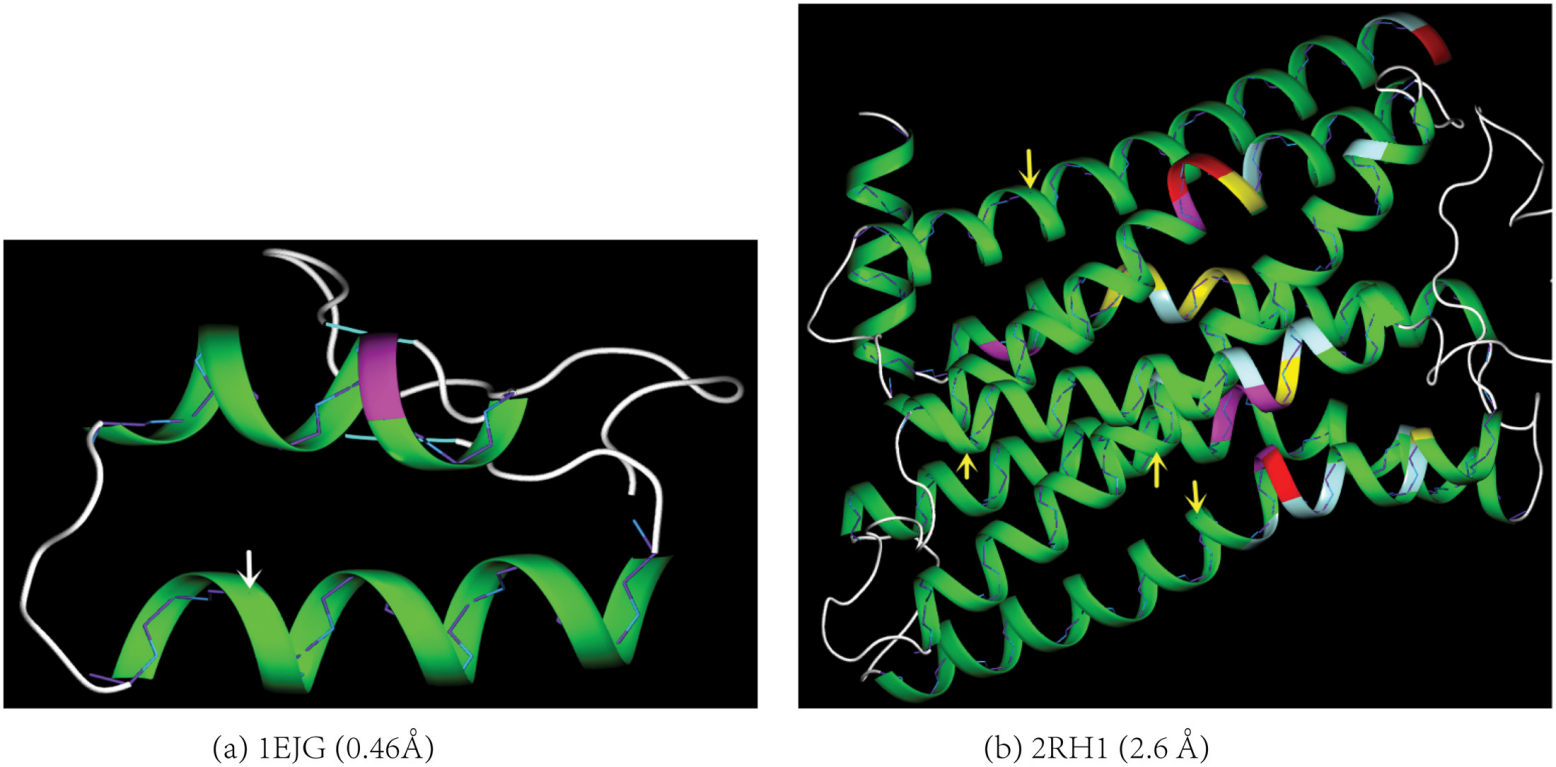
The model accuracy. The spatial differences between the backbone atoms and their closest points on the helix ribbon diagram generated by the model are barely visible (indicated by the arrows) for either (a) an ultra-high resolution protein structure (pdbid 1EJG) or (b) a medium-resolution structure (pdbid 2RH1). The backbones of the helices are shown in stick-and-ball with the diameter of the ball to be the same as the thickness of the ribbon. The C_α_ atoms are colored in cyan. A detachment occurs when a C_α_ atom is not positioned inside the ribbon diagram. The larger the difference is between a C_α_ atom and its closest point on the model, the larger its detachment from the diagram. The protein helix diagrams in both the main paper and Supporting Information (SI) are colored as follows according to residue’s helix score (Eq 2): 0.0–20.0 in green, 20.0–50.0 in celeste, 50.0–100.0 in yellow, 100.0–200.0 in magenta, > 200.0 in red. Except for Fig 1, all the figures in both the main paper and SI are prepared using our own molecular visualization program written in C++/openGL/Qt.

**Fig 3:**
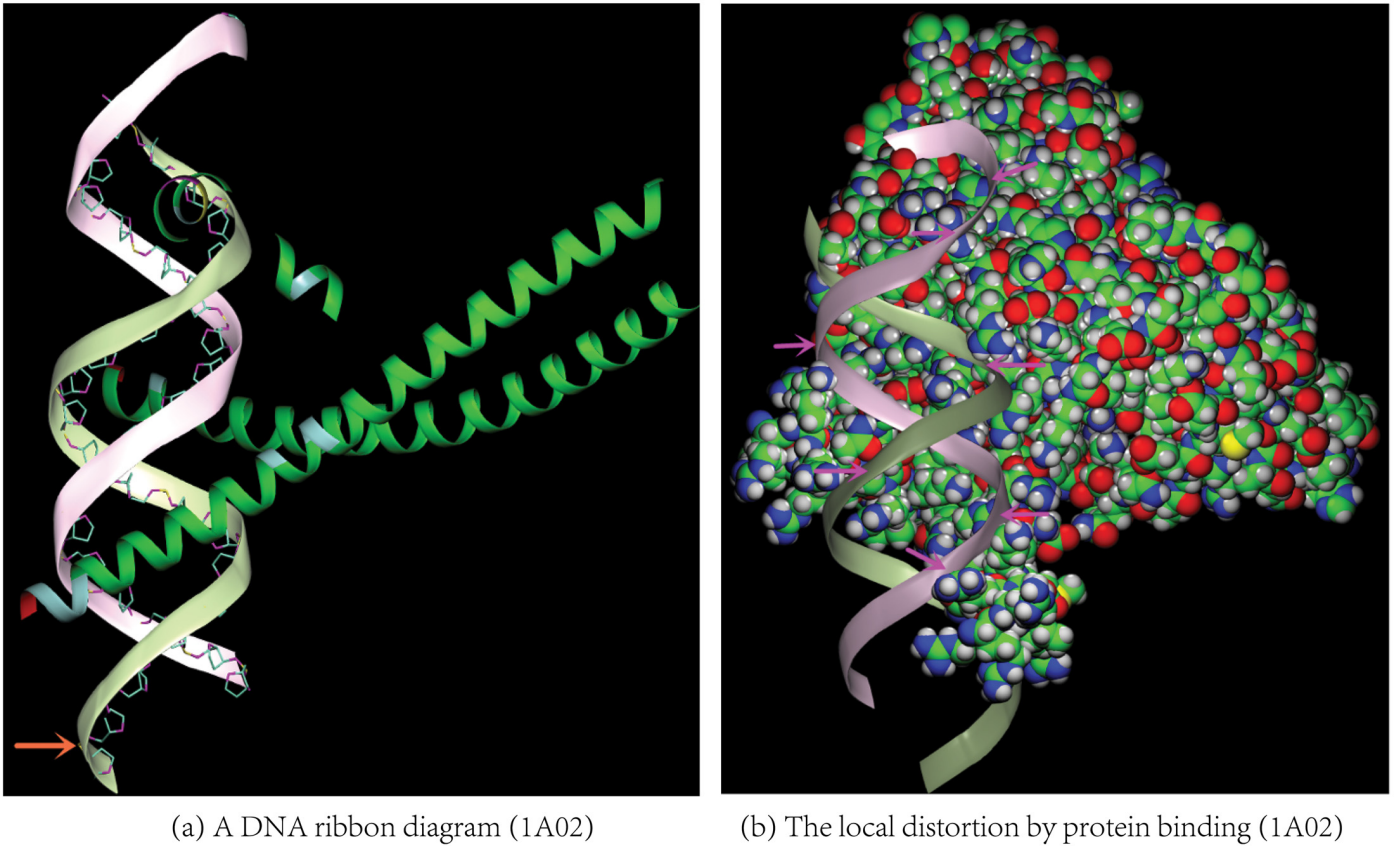
A DNA ribbon diagram and its local distortions. In (a) the differences between the DNA ribbon diagram generated by our helix model and the backbone P atoms (colored in maize) are visible for some nucleotides (indicated by an arrow) in a leucine zipper protein (pdbid 1A02). The protein ribbon diagram is colored according to residue’s helix score as in Fig 2. The residues with high helix scores are concentrated in the protein-DNA interface. For clarity neither protein loops nor β-strands are displayed in (a). In (b) the local distortions in the DNA ribbon diagram at the protein-DNA interface are indicated by the arrows. Here a local distortion means a twist away from an ideal helix ribbon diagram generated by a genuine helical curve (S4f, S4j and S5 Figs in the SI for additional examples of DNA ribbon diagrams). The protein atoms in (b) are colored as follows: H in gray, C in green, N in blue and S in yellow.

The application of the model to a set of 27,105 X-ray protein structures in the PDB with a resolution from 0.46Å to 3.5Å and less than 70% sequence identity confirms the model’s accuracy. Though the RMSDs (Δ_*i*_s in Eq 2) between the backbone C_*α*_ atoms and their closest points on the model range from 0.0 − 0.3Å for all the 262,266 DSSP-assigned protein helices, more than 95% of the helix residues have their Δ_*i*_s less than 0.08Å. As illustrated in Fig 2, the deviations between the experimental C_*α*_ positions and the model are barely discernible for the protein structures ranging from ultra-high resolution (pdbid 1EJG, 0.46Å), to medium resolution (pdbid 2RH1, 2.6Å), and to low resolution structures (pdbid 3ZC1, 3.3Å, S1 Fig in the Supporting Information (SI)). In general, the model accuracy is not affected by the residue’s helix score. For DNAs, the spatial difference between a backbone P atom and its closest point on the model could be large with a typical value from 0.0Å to 0.5Å(Fig 3a). Even with the relatively large difference between the model and the P atoms in DNAs, our model is superior to all the previous helix models for DNAs because the generated curves conform to a genuine helical curve much better than a series of splines generated by the previous models do (Please see S4f, S4j and S5 Figs in the SI for examples).

### 3.2 The helix score and the structural and functional significance of the model

Our model consists of a series of helical curves each one best fits the coordinates of a quadruple of backbone atoms. The individual curves could differ largely from each other depending on the extent of their deviations from a genuine helical curve. The deviation represents the structural difference among different biomolecular helices. They are described in our model by both the helical parameters and the RMSD Δ. Specifically we have defined a helix score for a protein helix residue (Eq 2) that includes both the deviation from the standard protein helix and the minimum RMSD achieved by the curve fitting algorithm. Either the score or the local deviations from a genuine helical curve could be visualized for individual protein residues or DNA nucleotides. A systematic survey over all the protein and DNA structures in the PDB is currently under way for the structural and functional significance of the helix score. As shown in Table 1 a preliminary study on a set of 3,446 x-ray structures with a resolution between 1.0Å–2.0Å and with less than 70% sequence identity has found a correlation between the residue’s helix scores and their locations in the proteins: the higher the score the higher probability of the residue being on a protein surface (Table 1).

**Table 1:** Helix score vs solvent accessible area (SAA) for proteins. The data are obtained on all the dssp [22] assigned helices on a set of 3,446 x-ray structures with a resolution between 1.0Å–2.0Å and with less than 70% sequence identity. There are 650,167 helix residues in total. In each column these residues are divided into two subsets, **S**_≤ *h*_*T*__ and **S**_> *h*_*T*__, according to a helix score threshold *h*_*T*_ with the residues in **S**_≤ *h*_*T*__ having *h*_*i*_ ≤ *h*_*T*_ while those in **S**_> *h*_*T*__ having *h*_*i*_ > *h*_*T*_. The parameter *λ* is computed by fitting a SAA histogram to an exponential function, *y* = *A*exp* λt* + *b*, where *A* and *b* are parameters and *t* and *y* are the variables. The parameter *μ* is the average of the SAAs for all the residues in **S**_≤ *h*_*T*__ or **S**_> *h*_*T*__. The unit of *μ* is Å^2^. As shown in the table *μ* increases but *λ* decreases with the helix score, and thus the higher the helix score the higher probability of the residue being on a protein surface.

Parameter	set of residues		set of residues		set of residues	
	**S**_≤20.0_	**S**_>20.0_	**S**_≤200.0_	**S**_>200.0_	**S**_≤2000.0_	**S**_>2000.0_
residues (%)	77.03%,	22.97%	87.42%,	12.58%	96.42%,	3.58%
*λ*	1.339,	0.895	1.314,	0.060	1.274,	0.018
*μ* (Å^2^)	19.55,	28.38	19.96,	32.70	20.96,	37.68

**Fig 4:**
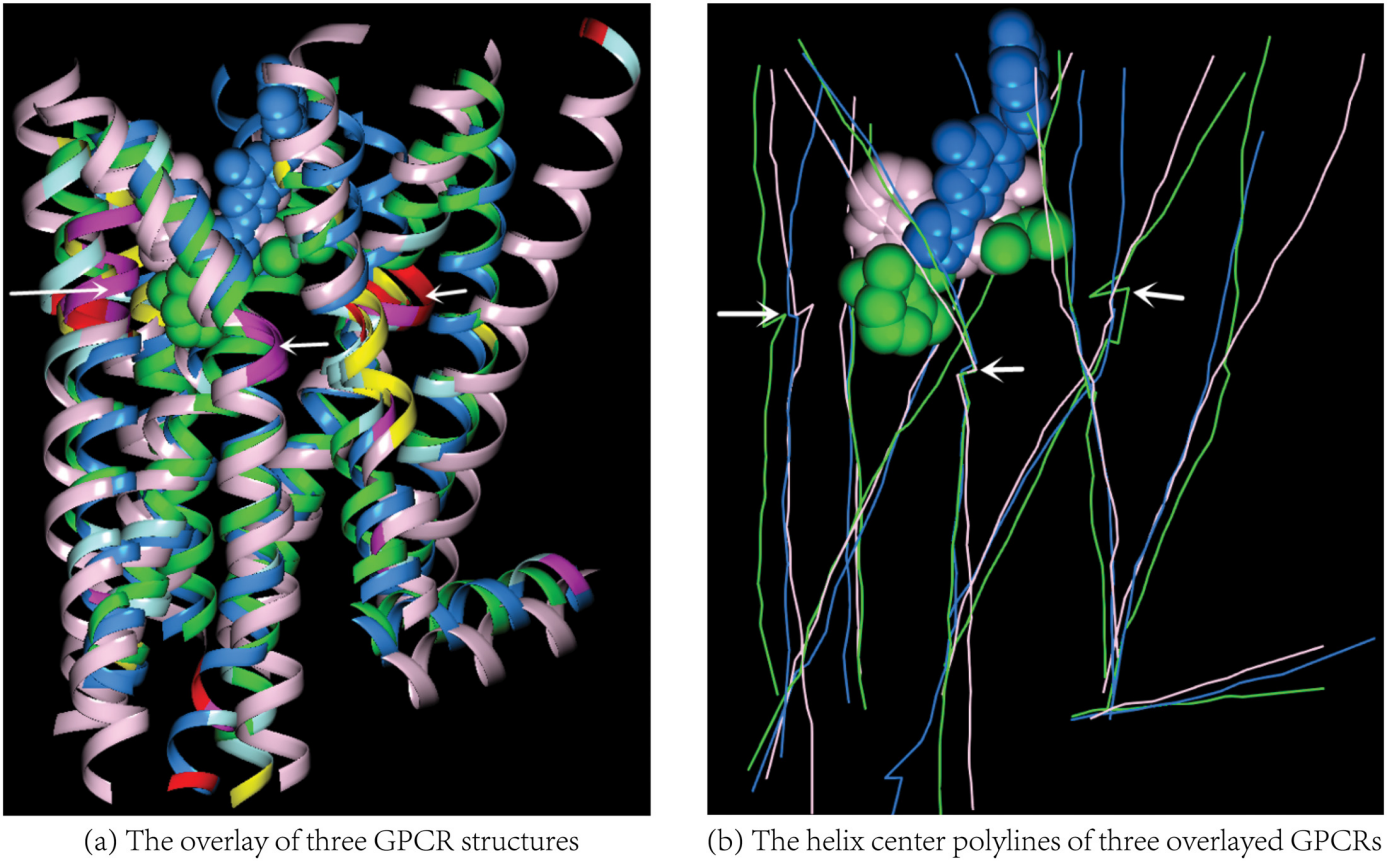
The deviations from the standard protein helix of the residues in the binding sites of three GPCR structures. In (a) the three GPCR structures (pdbid 1U19, 2RH1 and 3EML) are overlayed based on their sequence similarity [26]. The helix ribbon diagrams are colored as in Fig 2 except that the residues in 1U19, 2RH1 and 3EML with a helix score < 20.0 are colored respectively in green, classic rose and bright azure. The ligands and the helix center polylines in 1U19, 2RH1 and 3EML are similarly colored. In (b) each bend in a polyline indicates a deviation from the helix center polyline for a genuine helical curve, the latter is a straight line. Such bends are concentrated at the ligand binding sites.

The same study also shows that the helix residues in a protein-ligand binding site tend to have higher helix scores than the rest as illustrated in the three figures: Figs 3 and 4 and S1 Fig in the SI. The residues with high helix scores indicated by different colors are concentrated in the ligand binding sites where the ligand could be either a DNA molecule (Fig 3) or a compound (Fig 4) or other protein subunits (S1 Fig). Though it has been documented before [23–25] that the *π* and left-handed helices in proteins have higher probabilities to be in a ligand binding site, no scores have been proposed previously for a quantitative and consistent description of the distortions in all the three types of protein helices. One key advantage of a consistent helix score is that the scores for different homologs in the same protein family could be used to determine their structural similarity (conservation) and variation (Fig 4). In addition, with our model a polyline could be constructed for each biomolecular helix by connecting together the centers of the individual helical curves along either a protein helix (Fig 4b and S1 Fig) or a DNA strand (S4g and S5 Figs). For ease of reference, we call such a polyline *helix center polyline*. As illustrated in Fig 4b the abrupt changes (turns) in such a polyline occur often at a protein-ligand interface. Compared with the protein helices it is more tricky to quantify the deviations from a genuine helical curve for DNA helices because of their large structural variations. However our model is still able to provide a qualitative description for local deviations such as the twist away (Fig 3b) from an ideal helix ribbon diagram generated by a genuine helical curve (S4f, S4j and S5 Figs).

### 3.3 The application to helix visualization

**Fig 5:**
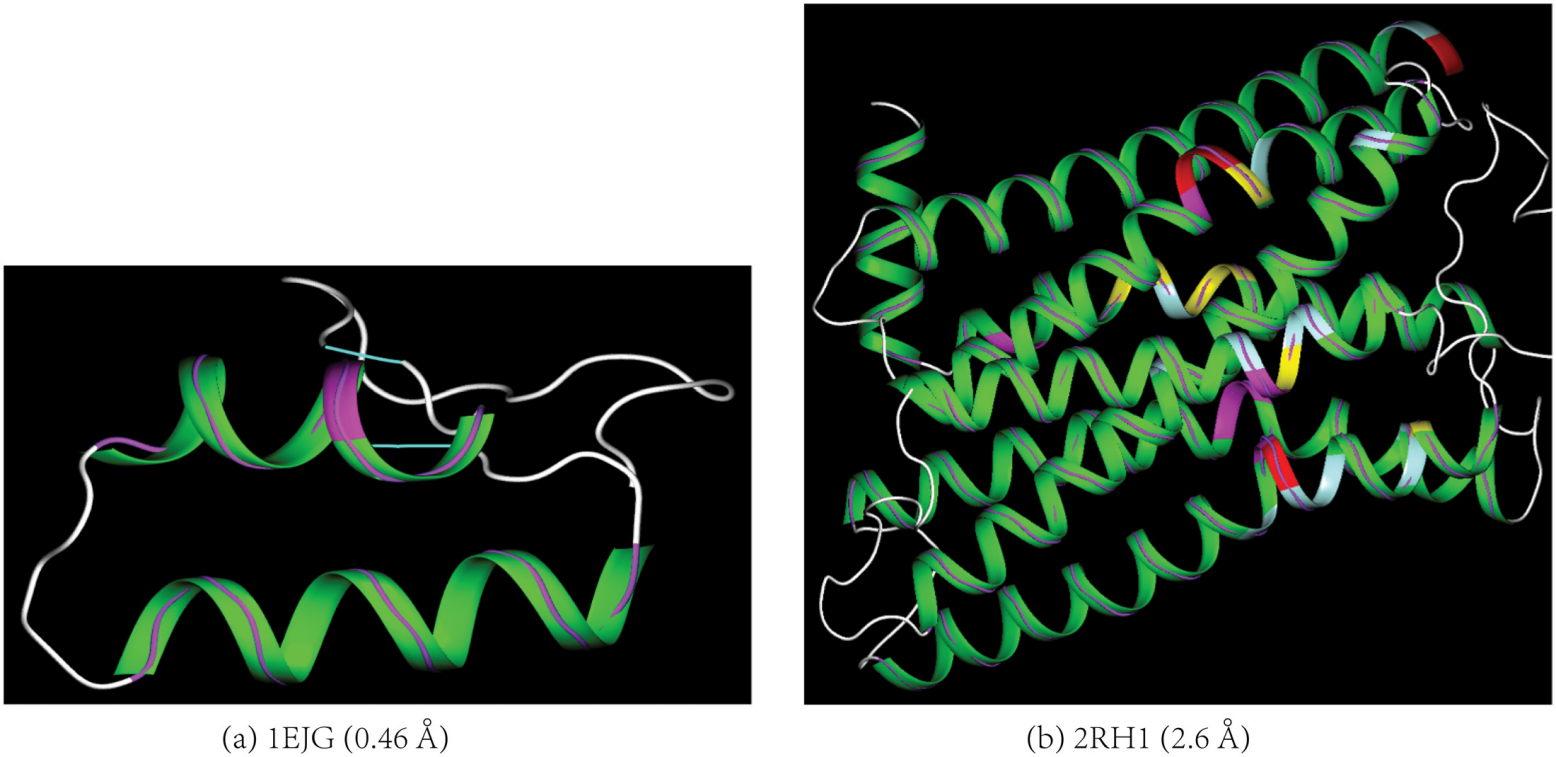
The helix ribbon diagrams generated by the model. The two figures illustrate the differences between the helix ribbon diagrams generated by the model and the helix ribbon diagrams generated using a series of cubic Hermite splines that pass through every backbone atom. The latter is colored in purple and drawn in sausage-shape and overlayed upon the former. As is clear by the comparison, the diagrams generated by Hermite splines are choppy while those by our model are much more smooth. The helix diagrams are colored according to the residue’s helix score as in Fig 2.

**Fig 6:**
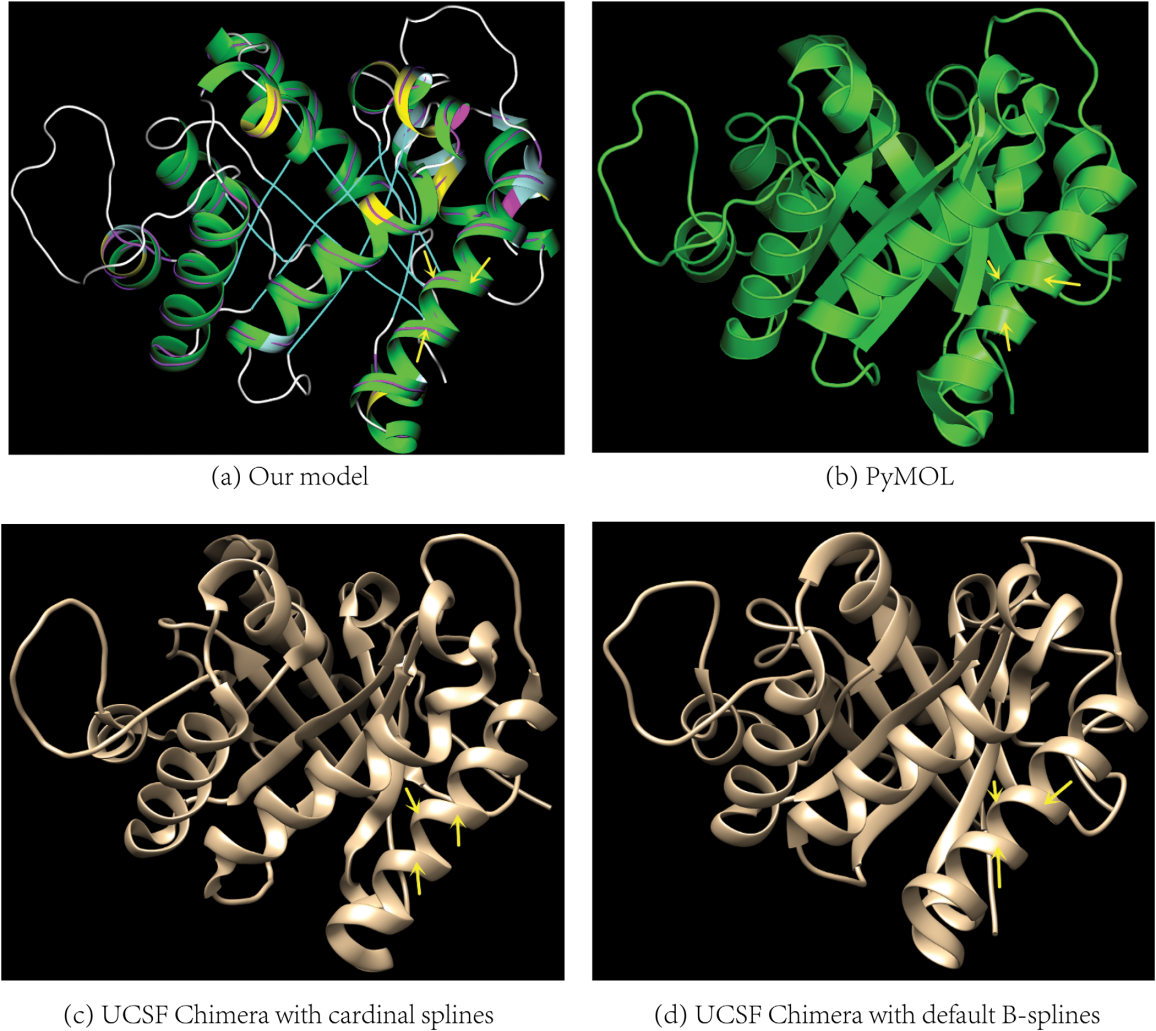
The comparisons of the helix ribbon diagrams by our model and two previous programs. The protein structure is 1TIM (pdbid) for which Prof. Jane Richardson had hand-drawn an iconic ribbon diagram [7]. The helices are oriented as close as possible to their orientations in her diagram (see S4 Fig). Shown in (a) is the diagram generated by our model, in (b) the diagram by the program PyMOL [18]. The diagrams in both (c) and (d) are drawn by the program UCSF Chimera [16]. The series of splines in (c) pass exactly through the C_α_ atoms and are computed using a series of quadruples of C_α_ atoms. Additional steps have been applied to smooth out the choppiness in (c). The splines in (d) are generated using a series of a quintuple of backbone C_α_ atoms. The side-chains in (d) could become detached. The choppiness in the PyMOL’s diagrams that pass exactly through the backbone atoms is less pronounced than those by other programs (see S3 Fig) but still visible upon a careful examination. It is likely that the original Hermite splines have been smoothed out to some extent in PyMOL.

It is a well-known problem in the modeling of a biomolecular helix as a series of low-degree splines that if the curves pass exactly through every backbone atom then the ribbon diagram generated by the model is choppy. On the other hand, if additional steps are applied or more than four backbone atoms are used for spline computation in order to smooth out the choppiness, then the side chains could become detached from the ribbon. The choppiness or detachment is inherent with a helix model that uses a series of splines where either the degrees of the splines are not high enough or the number of splines is not large enough for an accurate representation of a general helical curve that best fits the backbone atoms. Specifically the choppiness is due to nonsmooth changes in curvature along a biomolecular backbone while the detachment results from the spatial differences between the backbone atoms and the spline model. Though efforts have been made in the past [16, 18] to smooth out either the choppiness or detachment, either of them remains for the visualization of biomolecular helices in general and DNA helices in particular [17, 18] (please see S4 Fig). In our model the choppiness and detachment are simultaneously reduced to a great extent because the model itself is composed of a series of helical curves each one best fits a quadruple of backbone atoms. Specifically each helical curve has a unique curvature and the averaging process guarantees that the curvature for the final curve (the model) changes smoothly along a biomolecular backbone, consequently our model is able to almost eliminate the choppiness in protein helices (Figs 2, 4 and 5 and S1 Fig) and to greatly reduce it in DNA helices (S4f, S4g and S5 Figs). Similarly the averaging process keeps the distance between a backbone atom and its closest point on the model close to the minimum achieved by the curve fitting algorithm and thus the detachment problem is greatly alleviated. Fig 6 illustrates the differences between the ribbon diagram generated by our model and the diagrams by two previous programs. Please see the supporting information for the comparisons with four other molecular visualization programs (see S2 Fig). Our helix ribbon diagrams are most similar to the iconic ribbon diagram hand-drawn by Jane Richardson [7] (see S3 Fig). It is also rather similar to the ribbon diagram by UCSF Chimera [16] that uses a series of splines each one fits to *five* backbone atoms (Fig 6d). In contrast to the ribbon diagram by UCSF Chimera, our model minimizes the distance between a backbone atom and the ribbon diagram.

When four successive backbone P atoms are used to generate a ribbon diagram for a DNA helix, there often exist large variations among the diagrams for different DNAs and obvious deviations from a diagram that is generated using a single genuine helical curve (please see S5a Fig of the SI for an ideal helix ribbon diagram generated by our model using a single genuine helical curve). The variation and deviation are partly due to the small helix turn angle in a typical DNA helix. The turn angle for a typical DNA helix is 34°, about one third of the turn angle for a typical *α*-helix in proteins. If instead of using all the successive P atoms, when only the first atoms of every triple of consecutive P atoms are used to compute the model, the resulting helix ribbon diagram becomes much similar to both a protein helix diagram (S4h and S4j Fig) and an ideal helix ribbon diagram (S5a Fig). In this case the turn angle per atom is very close to that in a typical protein helix. However, such a DNA model has lower accuracy (S4i and S4j Fig).

### 3.4 The data set and the molecule visualization program

To evaluate the performance of our algorithm, we have downloaded from the current version of the PDB a set of 27,105 x-ray protein structures that have at most 70% sequence identity and each of them has at least one helix according to the PDB. From them a set of 3,287 high-resolution structures, 𝕊, each of them has at least three helices, a resolution ≤ 2.0Å and a R-factor ≤ 25.0% are selected for the statistical analyses of helical parameters and RMSDs.

We have implemented our helix model and its visualization in C++/Qt/openGL and included them as a module in our structure analysis and visualization program. The default values for the two parameters, *δ*_*r*_ and *δ*_*p*_, required for the computations of helix parameters *r*, *p* are set to 0.25 and the step size for both intervals, [*r*_*m*_ − *δ*_*r*_, *r*_*m*_ + *δ*_*r*_] and [*p*_*m*_ − *δ*_*p*_, *p*_*m*_ + *δ*_*p*_], is 0.01. The program is written in Qt5.3/openGL4.3/GLSL4.3 and is available upon request.

## Supporting Information

S1 Fig The model accuracy for low resolution protein structures. As illustrated in S1 Fig the accuracy of our model is not affected by the resolutions and R-factors of protein x-ray structures. The side chain detachment from the ribbon diagram for a low resolution protein structure. The protein is a homo-octomer composed of eight identical subunits (pdbid 3ZC1, 3.3Å). The protein backbone is shown in stick-and-ball with the C_*α*_ atoms in cyan. A detachment occurs when a C_*α*_ atom is not positioned inside the ribbon diagram. No detachments are discernible in this helix ribbon diagram. The helix center polylines are shown in orange. The diagrams are colored as in Fig 2 of the main paper.
(EPS)

S2 Fig The comparisons with previous molecular visualization programs. In addition to the comparisons with the two previous molecular visualization programs (PyMOL and UCSF Chimera) described in the main paper, we have also compared the helix ribbon diagram generated for 1TIM (pdbid) by our model with those by four other molecular visualization programs (S2 Fig) and with the iconic diagram hand-drawn by Jane Richardson (S3 Fig)The protein structure is 1TIM (pdbid). Shown in (a, b, c, d) is, respectively, the diagram generated by Jmol [20], VMD [15], BALLView [19], and Molsoft [12]. The arrows point to the locations where the diagrams are choppy.
(EPS)

S3 Fig The ribbon diagram for 1TIM hand-drawn by Jane Richardson. With the kind permission from Prof. Jane Richardson.
(EPS)

S4 Fig The DNA helix ribbon diagrams by five molecular visualization programs and their comparisons with the diagrams by our model. Compared with protein helices DNA helices have larger variations among themselves and larger deviations from a genuine helical curve. This figure shows the DNA ribbon diagrams by five previous molecular visualization programs and their comparisons with the ribbon diagrams generated by our model. The structure is a leucine zipper protein (pdbid 1A02) with a bound dsDNA. Figures **a, b, c, d, e, f** and **g** are, respectively, the ribbon diagrams by UCSF Chimera [16], PyMol [18], Molsoft [12], BALLView [19], VMD [15] and the two ribbon diagrams generated by our model using four consecutive P atoms. The two polylines in (**g**) are the helix center polylines. The two figures, **h** and **i**, are respectively the helix ribbon diagram generated by our model using a series of quadruples of atoms with each atom is the first of a triple of consecutive P atoms along a DNA strand, and its overlay with the ribbon diagram generated using successive four P atoms. The two figures **i, j** are the same except that the heavy atoms are displayed in the latter.
(EPS)

S5 Fig The helix ribbon diagrams for two B-DNAs. This figure shows a DNA ribbon diagram generated by our model for a theoretical B-DNA model with its first strand to be constructed using a set of standard parameters for a B-DNA molecule and a diagram for an experimental X-ray B-DNA structure. The helix center polyline is a straight line for the first stand of the theoretical B-DNA model but base-pairing requirement forces its second strand to deviate from a genuine helical curve. Figure (**a**) is a ribbon diagram generated by our model for a B-DNA model with the first strand (colored in classic rose) constructed using a set of standard B-DNA parameters. Base-paring requirement forces the second stand (colored in light olive) to deviate from a genuine helical curve. The helix center polyline is a straight line for the first strand. Shown in (**b**) is a ribbon diagram for an experimental x-ray B-DNA structure (pdbid 9BNA).
(EPS)
